# Acute Suppurative Thyroiditis in Childhood: An Atypical Presentation

**DOI:** 10.7759/cureus.55275

**Published:** 2024-02-29

**Authors:** Beatriz Câmara, Catarina Andrade, Andreia Forno, Marta Lopes, Carla Pilar

**Affiliations:** 1 Pediatrics, Hospital Dr. Nélio Mendonça, Funchal, PRT; 2 Pediatric Neurology, Hospital Dr. Nélio Mendonça, Funchal, PRT; 3 Otolaryngology, Hospital Dr. Nélio Mendonça, Funchal, PRT; 4 Pediatric Surgery, Hospital Dr. Nélio Mendonça, Funchal, PRT

**Keywords:** thyroid abscess, endocrine emergency, recurrent neck mass, pyriform sinus fistula, acute suppurative thyroiditis

## Abstract

Neck stiffness is a common clinical sign in children presenting to the emergency department that may indicate a wide variety of diagnoses. Acute suppurative thyroiditis (AST) is an infection of the thyroid gland caused by a bacterium, virus, or, less commonly, fungus. It presents as an acute or subacute development of an anterior cervical mass, with associated inflammatory signs. The pressure upon neck muscles may be reflected as a limitation of cervical mobility. AST is often preceded by an upper respiratory tract infection, and pyriform sinus fistula is the most common predisposing factor. It is particularly uncommon in the pediatric age, with limited cases reported in the literature. Therefore, a heightened suspicion is required for proper diagnosis and timely intervention, due to its high mortality. Prompt treatment with broad-spectrum parenteral antibiotic therapy and drainage is mandatory to prevent the numerous complications associated, namely, mediastinitis and sepsis.

We report the case of a two-year-old female child admitted with a two-week history of intermittent high-grade fever and sore throat, followed by prostration and limitation in neck movement on admission. Examination revealed neck stiffness with positive Kernig and Brudzinski signs. The laboratory tests showed elevated inflammatory parameters. Cranial computed tomography (CT) scan and lumbar puncture were normal. On day 2 after admission, an anterior cervical mass with slight signs of fluctuation was detected. Ultrasound was compatible with a hemorrhagic/overinfected thyroid nodule, and the patient was started on broad-spectrum antibiotics. Due to clinical worsening, a cervical CT scan was performed, which documented a thyroid abscess with extension into the retropharyngeal space. She underwent surgical drainage, and *Streptococcus anginosus* and mixed anaerobes were isolated, sensitive to ongoing antibiotherapy. On multidisciplinary follow-up, an esophageal barium study, laryngoscopy, and cervical magnetic resonance imaging (MRI) were performed, revealing no anatomical defects.

AST is a rare disease in children, but potentially fatal, so its early recognition and treatment are essential. We aim to draw attention to this disease and its differential diagnosis to reduce the associated morbimortality.

## Introduction

Neck stiffness is defined as an abnormal position of the neck or a normal position associated with a limited range of motion. It is a common clinical sign in children presenting to the emergency department and may represent a broad range of conditions, from benign to life-threatening. If fever is present, particularly in an ill-appearing child, the possibility of meningitis must be considered. However, other neck infections may present with fever and meningismus, such as cervical lymphadenitis, retropharyngeal abscess, septic thrombophlebitis, and thyroid infections [1].

Acute suppurative thyroiditis (AST) is an unusual type of head and neck infection in children, with few cases described worldwide [2,3]. The thyroid gland is relatively resistant to infection because of its high inner concentration of iodine and hydrogen peroxide necessary for thyroid hormone synthesis. Additionally, the presence of a thyroid capsule contributes to the anatomical isolation of the gland from other neck structures [4]. Nevertheless, hematogenous or direct spread, pre-existing thyroid disorders, and immunosuppression may predispose the gland to become infected [2]. In children, this is often secondary to local congenital anatomic defects, such as a thyroglossal duct remnant. In particular, pyriform sinus tracts, once thought to be rare anomalies, are being identified with increasing frequency [4,5]. Of all thyroid diseases, AST has an estimated incidence of 0.1-0.7%, with a recurrence rate of 16% in fully recovered cases. It is crucial to make a timely diagnosis due to its mortality of 12% or higher. Most cases occur under the age of 40 years, with a slight female predominance [6].

We report a case of a child admitted to a pediatric ward due to fever, prostration, and limitation of cervical mobility, in which the diagnosis of AST was particularly challenging.

## Case presentation

A two-year-old girl with a previous medical history of failure to thrive presented to the emergency department with a two-day history of refusal to move the neck, refusal to eat (with tolerance for liquids only), prostration, and groaning. In addition, she had intermittent high-grade fever and sore throat two weeks prior to this presentation. She had been treated with oral amoxicillin for acute tonsillitis in the previous three days, without improvement. Her sisters had been diagnosed with herpangina. She had no other relevant epidemiological context. She was vaccinated according to the Portuguese National Vaccination Program against 13-serotype *Streptococcus pneumoniae*, *Neisseria meningitidis* B, *Neisseria meningitidis* C, and *Haemophilus influenzae* type b disease. She was the fourth child of a healthy non-consanguineous couple with no relevant family history, namely, thyroid diseases, cancer, or autoimmune disorders.

On initial assessment, she was pale, with a constant moan, and ill-appearing. She exhibited significant cervical stiffness upon passive mobilization, and Kernig and Brudzinski signs were positive. Due to a fixed posture, the anterior cervical region was difficult to visualize at admission, and the cervical mass was not detected. There were no adenopathies on palpation of the lateral cervical and supraclavicular regions. There was no trismus, and the observation of the oropharynx did not reveal any signs suggestive of abscess, namely, uvula deviation or bulging palate. There were no other pathologic findings on physical examination, such as petechiae, hemorrhagic suffusions, or other rashes.

Considering the possibility of meningitis, blood tests and a cranial computed tomography (CT) scan were requested, in order to exclude intracranial hypertension and other formal contraindications to the performance of a lumbar puncture. A complete blood count showed a hemoglobin level of 10.3 g/dL and white blood cell counts of 21900 cells/mm^3^ with 75% neutrophils and 19% lymphocytes. The C-reactive protein (CRP) level was increased to 323.7 mg/L (normal range: <5 mg/L) and procalcitonin (PCT) to 0.46 ng/mL (normal range: <0.05 ng/mL) (Table 1). The cranial CT scan was normal and a lumbar puncture was performed. She was treated for suspected meningitis with ceftriaxone 100 mg/kg/day and vancomycin 60 mg/kg/day and was admitted for monitoring. Cerebrospinal fluid analysis was normal (Table 2).

**Table 1:** Evolution of laboratory parameters throughout hospitalization PT: prothrombin time; INR: international normalized ratio; aPTT: activated partial thromboplastin time; CRP: C-reactive protein; PCT: procalcitonin; ESR: erythrocyte sedimentation rate; ALT: alanine transaminase; AST: aspartate aminotransferase; TSH: thyroid-stimulating hormone; T3: triiodothyronine; T4: thyroxine

Laboratory parameters (reference range)	Hospitalization				One month after discharge
	Before surgery		After surgery	At discharge	
	On admission	Day 2	Day 7	Day 14	
Leukocytes (4500-17000/μl)	22000/μl	24600/μl	11700/μl	10800/μl	8300/μl
Neutrophils (1500-5500/μl)	16600/μl	20300/μl	4100/μl	4300/μl	2500/μl
Lymphocytes (2000-34000/μl)	4100/μl	3300/μl	6600/μl	5400/μl	5000/μl
Hemoglobin (10.1-12.7 g/dL)	10.1 g/dL	10.0 g/dL	13.0 g/dL	11.4 g/dL	11.7 g/dL
Platelets (144000-445000/μl)	501000/μl	572000/μl	446000/μl	337000/μl	250000/μl
PT (9.4-12.5s)	13.6s	13.3s			
INR (0.9-1.2)	1.17	1.14			
aPTT (25-37s)	28s	23s			
Glucose (60-100 mg/dL)	102 mg/dL	560 mg/dL		80 mg/dL	
Urea (8-50 mg/dL)	26 mg/dL	121 mg/dL	26 mg/dL	25 mg/dL	51 mg/dL
Creatinine (0.3-0.7 mg/dL)	0.23 mg/dL	27 mg/dL	0.28 mg/dL	0.29 mg/dL	0.28 mg/dL
Sodium (136-15g mEq/L)	139 mEq/L	0.22 mg/dL	136 mEq/L	136 mEq/L	139 mEq/L
Potassium (3.5-5.1 mEq/L)	4.5 mEq/L	140 mEq/L	4.7 mEq/L	4.9 mEq/L	4.4 mEq/L
Calcium (8.8-10.8 mg/dL)	9.07 mg/dL		9.05 mg/dL	9.1 mg/dL	10.48 mg/dL
Phosphorus (3.4-6.7 mg/dL)			3.4 mg/dL	4.5 mg/dL	4.9 mg/dL
AST (15-60 U/L)	27.0 U/L		17 U/L	23 U/L	19 U/L
ALT (17-63 U/L)	40.0 U/L		23 U/L	24 U/L	28
CRP (<6.10 mg/L)	323.76 mg/L		17.2 mg/L	6.58 mg/L	
PCT (high risk of sepsis >2 ng/mL)	0.46 ng/mL	226.14 mg/L			
TSH (0.30-4.70 μUI/mL)			0.06 μUI/mL	3.5 μUI/mL	4.24 μUI/mL
T3 (2.0-4.4 ng/dL)		0.6 μUI/mL	2.0 ng/dL	2.2 ng/dL	3.6 ng/dL
T4 (0.6-1.7 ng/dL)			1.6 ng/dL	0.78 ng/dL	1.4 ng/dL

**Table 2:** Complementary evaluation ANA: anti-nuclear antibodies; ENA: extractable nuclear antigen; CMV: *Cytomegalovirus*; EBV: Epstein-Barr virus; VCA: viral capsid antigen; EBNA: Epstein-Barr nuclear antigens; HIV: human immunodeficiency virus; IgG: immunoglobulin G; IgM: immunoglobulin M

Etiological investigation			
Hemocultures	Negatives		
Cerebrospinal fluid analysis	Color		Clear
	White blood cell count (0-20 mm^3^)		<1 mm^3^
	CSF glucose (60-80 mg/dL)		73 mg/dL
	CSF protein (15-45 mg/dL)		8.4 mg/dL
	Microbial examination		No microorganism
	Cultural		Negative
Pus cultures	Bacterial	Positive for *Streptococcus anginosus* sensible to penicillin G, ampicillin, and ceftriaxone	
		Positive for anaerobic gram-negative bacilli	
	Fungal	Negative	
	Mycobacterial	Negative	
ANA	Negative (AC-0)		
ENA screen (negative <1.0)	0.0 (negative)		
Ac. Anti-dsDNA IgG (negative <30.0 IU/mL)	8.5 IU/mL (negative)		
EBV EBNA-IgG antibody (positive >1.0 S/CO)	11.2 (positive)		
EBV VCA-IgG antibody (negative <0.75 S/CO)	35.5 (positive)		
EBV VCA-IgM antibody (negative <0.5 S/CO)	0.1 (negative)		
CMV IgG antibody (positive >15 UA/mL)	229.4 UA/mL (positive)		
CMV IgG antibody avidity assay (high avidity >60%)	73.6%		
CMV IgM antibody (negative <0.85 S/CO)	0.4 S/CO (negative)		
HIV antigen/antibody test	Negative		
Complement component 3 (reference range: 90-180 mg/dL)	139 mg/dL		
Complement component 4 (reference range: 10-40 mg/dL)	19.6 mg/dL		
Immunoglobulin G (reference range: 420-1200 mg/dL)	678 mg/dL		
Immunoglobulin M (reference range: 18-150 mg/dL)	74.7 mg/dL		
Immunoglobulin A (reference range: 45-200 mg/dL)	86.3 mg/dL		

**Figure 1:**
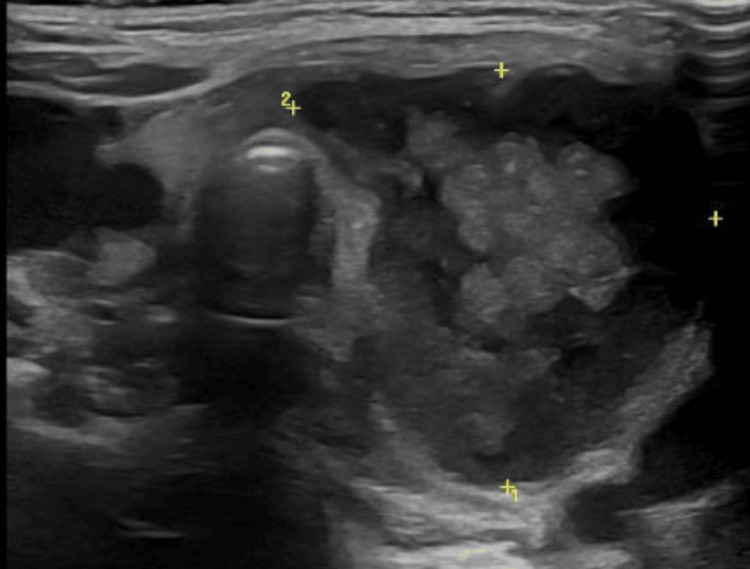
Thyroid ultrasound Thyroid ultrasound revealed a lobed heterogeneous iso- and hypoechoic mass, sized 2.8x3.0x3.7 cm, at the left thyroid lobe.

On the second day, an anterior cervical mass of rubbery consistency, painful on palpation, with slight signs of fluctuation was detected. The swelling moved with deglutition. She had no signs of airway compression. Considering the possibility of an infected thyroglossal canal cyst, a cervical ultrasound was requested. This exam demonstrated a lobed heterogeneous iso- and hypoechoic mass, sized 2.8x3.0x3.7 cm, at the left thyroid lobe, compatible with a hemorrhagic/overinfected thyroid nodule, with no signs of airway compression (Figure 1). Her thyroid function testing was normal, with free thyroxine of 1.9 ng/dL (normal range: 0.3-4.7 ng/dL) and thyroid-stimulating hormone of 0.6 μUI/L (normal range: 0.4-4.9 μUI/L). After a multidisciplinary discussion involving Endocrinology, Otorhinolaryngology, and Pediatric Surgery Departments, a conservative approach was decided. Clindamycin was added, so a triple empiric antibiotic regimen was maintained, with an indication for thyroid nodule cytology after stabilisation. Intravenous methylprednisolone was given to decrease subglottic edema and thyroid inflammation.

**Figure 2:**
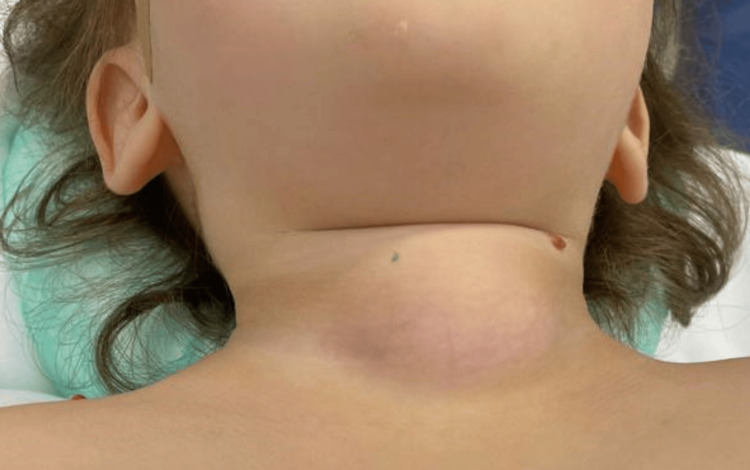
Cervical mass with inflammatory signs An anterior cervical mass of rubbery consistency with signs of fluctuation was detected on day 2 after admission.

**Figure 3:**
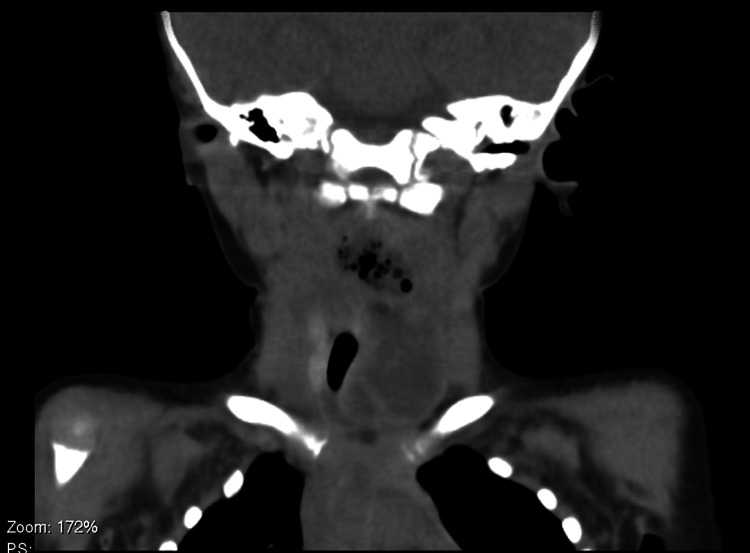
Cervical CT scan: coronal view A cervical CT scan revealed a thyroid abscess, measuring 4.3x1.5x2.4 cm, with interior gas bubbles suggestive of anaerobic infection. CT: computed tomography

**Figure 4:**
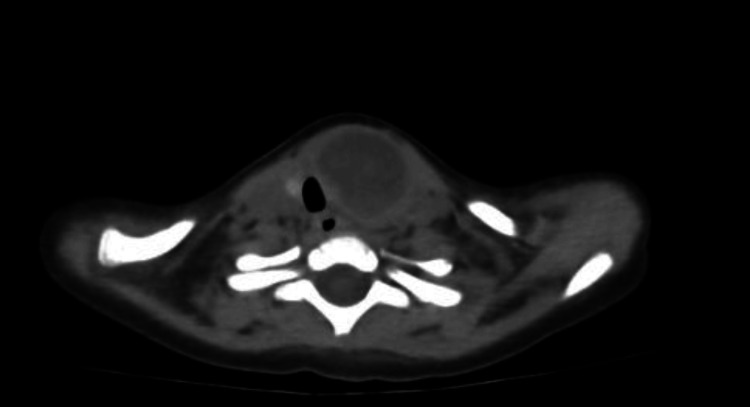
Cervical CT scan: axial view A cervical CT scan revealed a collection that caused the left deviation of the trachea and extended into the retropharyngeal space. CT: computed tomography

On day 3 of admission, despite being afebrile, there was a worsening of the general condition. She developed signs of airway compromise, namely, muffled voice, profuse sialorrhea, and choking on oropharyngeal secretions and liquids. Upon observation, there was an increase in the dimensions of the cervical lesion (Figure 2). An urgent cervical CT scan revealed a complicated thyroid abscess extending into the retropharyngeal space, with 4.3x1.5x2.4 cm. The lesion had gas bubbles inside and caused a left deviation of the trachea, with minimal obliteration of its lumen (Figure 3 and Figure 4). 

**Figure 5:**
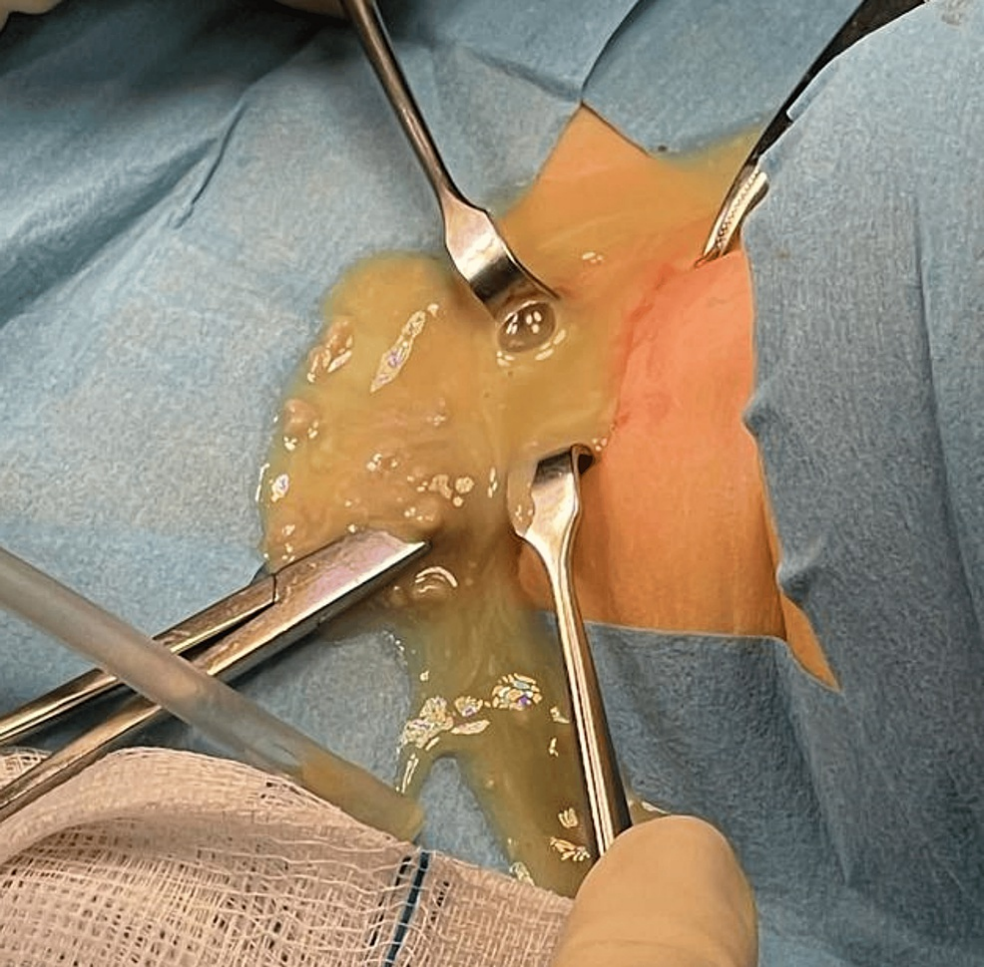
Surgical drainage of thyroid abscess

Considering she was already under broad-spectrum antibiotics, with adequate coverage for most aerobic and anaerobic bacteria, surgical drainage of the multiloculated abscess was performed, releasing about 9 mL of thick yellow drainage (Figure 5). She required a surgical washout and drain tubes were inserted. Afterwards, she was admitted to the pediatric intensive care unit, where she remained for four days, with good clinical-analytical progress. Immunological and autoimmunity studies as well as viral serologies were requested, in order to clarify the etiology and predisposing factors, such as immunosuppression, that were all normal. Intraoperative cultures isolated *Streptococcus anginosus* and mixed anaerobes, sensitive to penicillin, amoxicillin, and ceftriaxone. Histology was normal. Three blood cultures obtained prior to antimicrobial therapy were negative (Table 2).

Intravenous antibiotics were continued for 14 days, after which she was discharged. Antibiotic coverage was continued at home with amoxicillin-clavulanic acid and clindamycin for a total of 21 days. She has now been followed for one year without recurrence, always in a euthyroid state. The follow-up thyroid ultrasound identified a heterogeneous formation, with predominantly cystic content, in relation to the residual collection. A barium esophagogram, flexible nasoendoscopy, and cervical magnetic resonance imaging (MRI) were performed more than one month after AST resolution but failed to identify a pyriform sinus fistula or any other anatomical defect. Further investigations, including studies of potential immunodeficiency disorders, did not reveal a definitive source of infection.

## Discussion

AST is a rare and potentially life-threatening disease. In immunocompetent patients, this condition is most often encountered in those with congenital anomalies, such as a pyriform sinus fistula [7]. The left lobe is predominantly affected due to the unequal development of the brachial derivatives that result in the pyriform tract fistula [8]. In accordance with our case report, AST is often preceded by an upper respiratory tract infection. This is due to an altered local immune response and bacterial overgrowth of indigenous flora that spreads by a communicating fistula to the thyroid gland [9]. The most common pathogens causing AST are bacteria, particularly gram-positive ones, including staphylococcal and streptococcal species. Furthermore, fungi, viruses, and parasites have also been involved [10]. In our patient, intraoperative cultures were positive for *Streptococcus anginosus* and mixed anaerobes. According to the literature, polymicrobial infection (two to five organisms per abscess) occurs in 30% of patients [2].

Most cases of AST present with a painful swelling with inflammatory signs in the anterior cervical region that moves on swallowing. Neck pain reduces with flexion and worsens with neck hyperextension, and the pressure upon the neck muscle reflects in limitations of cervical mobility [2]. This was particularly pronounced in our patient, where neck stiffness delayed the final diagnosis due to consideration of other more frequent causes of meningismus. Further manifestations include fever, sore throat, dysphagia, and dysphonia. The diagnosis may be delayed from days to months (with a mean of 18 days) after the initiation of symptoms [11]. Abnormal laboratory findings include leukocytosis and elevated inflammatory markers [12]. As demonstrated by our patient, most individuals are euthyroid at admission [8,9,12]. Nevertheless, thyrotoxicosis caused by follicular disruption and hypothyroidism may be identified in 12% and 17% of cases, respectively [6].

Imaging can help establish an early diagnosis and assist in differentiation from other more common conditions. As the ultrasound does not carry ionizing radiation and is a simple and convenient process, it was the initial imaging test [10]. Ultrasonographic findings assist in differentiating solid versus cystic or mixed lesions, with or without air [9]. Sonography also allows radiographically guided drainage of a thyroid abscess if present. A CT scan is advisable if the clinical course suggests an extension of the infection to other structures, as it provides a more accurate mapping than ultrasound [3].

Prompt treatment is mandatory to prevent the numerous complications associated with AST. These include airway obstruction, sepsis/multiorgan failure, extension or rupture of the abscess with mediastinitis, pericarditis, or thrombophlebitis, paralysis of the vocal cord, or compression of sympathetic chain with subsequent Horner syndrome [3]. Given the destruction of local tissue in AST, thyrotoxicosis may also develop [13].

Due to its rarity, clinical guidelines are lacking, and treatment recommendations are drawn from retrospective case reports and case series. Treatment choice is determined by the stage of AST and involves supportive care and broad-spectrum parenteral antibiotic therapy. The initial antibiotic choice should be based on the patient's medical history, predisposing factors, the severity of infection, and regional antimicrobial resistance patterns [14]. If the patient is stable and immunocompetent, first-line empirical therapy should include antibiotics with activity against gram-positive bacteria, namely, penicillin/β-lactamase inhibitors or second-generation cephalosporins, associated with vancomycin if methicillin-resistant *Staphylococcus aureus* is suspected [15]. Antibiotics should be adjusted afterwards according to cultural and sensitivity results and continued for 14 days or until complete clinical resolution [15]. In addition, beta-blockade to control the peripheral effects of thyroid hormones may be considered [10]. If fluctuation or sonographic evidence of an abscess is present, drainage is generally necessary [2]. Drainage through needle aspiration is increasingly common as the primary treatment, due to its safety and effectiveness. However, needle aspiration may not be sufficient in deteriorating patients, and for that reason, surgery was required in our patient. If extensive disease is present, or if it is refractory to incision and drainage, hemi- or total thyroidectomy may become necessary [15]. Post-surgical care should include assessment of active hemorrhage, airway compromise due to tissue edema and seroma, and damage to tissues in the area, such as the parathyroid glands and the recurrent and superior laryngeal nerves [16]. It should also include monitoring of thyroid function, due to the manipulation of thyroid structures during surgery, with follicle disruption. Specimens should be obtained for Gram stain and microbiological studies to assist in the proper antimicrobial therapy [3]. Moreover, cytology should be performed to exclude malignancy [2].

Given the possibility of recurrence, long-term follow-up is advisable. After successful resolution of thyroid abscess, it is important to rule out pyriform sinus anomalies, but the diagnosis and management are challenging [17]. During the acute inflammatory phase, swelling of the mucosa and adjacent tissue can cause partial or complete obstruction of the fistula tract [18,19]. Hence, a barium swallow test is recommended four to six weeks after the inflammation subsides [5]. However, negative findings on barium swallow testing do not rule out a tract, and direct laryngoscopy, CT with contrast, or MRI should be performed. When a fistula is identified, a range of treatment options can be considered, including endoscopic cauterization or complete surgical excision of the fistulous tract [19]. Moreover, thyroid function should be repeated in four to six weeks or if symptoms of hypothyroidism develop [17].

Lafontaine et al. [11] did a systematic review of 148 articles published over the past 20 years that described a total of 200 cases of pediatric and adult AST. Of those, only 32 cases were children. There was a wide range of ages included (1-87 years old), with a female predominance. The most common symptoms were neck pain (89%) and fever (82%), which presented with a median duration of seven days before presentation. Our patient presented symptoms of intermittent fever and odynophagia for two weeks before admission. However, it is unclear whether these were the initial incipient symptoms of suppurative thyroiditis or of an upper airway infection that preceded its development. As with our patient, neck pain was particularly exuberant. Inflammatory markers were elevated in the majority of patients with bacterial AST, and the diagnoses were established through ultrasound and CT in 56% and 74% of the cases, respectively. The majority of bacterial AST were due to gram-positive bacteria, with the left thyroid lobe most frequently affected, similar to our report. In contrast, a high proportion of patients developed hyperthyroidism (42%) during disease management. Regarding treatment, the approach was highly heterogeneous: 32% were managed with antibiotics and a single needle aspiration, 3% required multiple needle aspirations, and 13% started with antibiotics and needle aspiration but subsequently required surgery. In this context, incision and drainage was also the most common procedure.

She et al. [16] retrospectively reviewed the clinical data of 18 children treated between 2009 and 2022 in a tertiary hospital in China. Our patient was younger than most pediatric cases reported in the literature. In this review, the median age at diagnosis was 7.8±3.8 years. Similarly, the main clinical manifestations were fever (88.9%), neck pain (100%), and neck mass (100%), the majority involving the left thyroid lobe. Importantly, as it was in our case, three patients were misdiagnosed at the initial presentation: two as subacute thyroiditis (SAT) and one as infectious mononucleosis. This fact underscores the importance of maintaining a higher clinical suspicion early on. Among the 13 patients who completed thyroid function tests, the majority were euthyroid. The most frequently identified agents were gram-positive bacteria that grew exclusively in pus cultures. For this reason, second-generation cephalosporin and penicillin/β-lactamase inhibitors were the empirical choices in most cases, given either alone (16.7%) or in association with ultrasound-guided needle aspiration (38.9%) or surgical incision and drainage (38.9%). Of note, the authors highlight two cases whose clinical condition worsened after glucocorticoids. This could also be the case of our patient, who was initially given methylprednisolone in order to reduce the inflammation and edema of the airway, but whose symptoms aggravated subsequently. Follow-up was conducted in 15 of the 18 patients, for one month to 11 years, during which three patients relapsed. Congenital anomalies were detected in seven children (pyriform sinus fistula, branchial cleft cyst, and thyroglossal duct cyst), and one child had a metabolic disorder with chronic neutropenia.

## Conclusions

Suppurative thyroiditis with the formation of an intrathyroidal abscess is an uncommon pediatric emergency. In the early stages, AST may present with atypical symptoms that overlap with other more common disorders, as was shown in the present case where misdiagnosis with meningitis occurred due to neck stiffness. Therefore, the clinician needs to consider AST in the differential diagnosis of a patient with neck pain, neck mass, and/or fever, who doesn't respond to the initial management. Congenital anatomical abnormalities must be sought and addressed in all children with AST. In particular, a left-sided intrathyroidal abscess should immediately arouse suspicion of a pyriform sinus tract. The present clinical case aims at a greater recognition of this diagnostic entity and correct clinical management to reduce the associated morbimortality.
